# Should the Mediterranean diet be recommended for inflammatory bowel diseases patients? A narrative review

**DOI:** 10.3389/fnut.2022.1088693

**Published:** 2023-01-10

**Authors:** Alicja Ewa Ratajczak, Stefano Festa, Annalisa Aratari, Claudio Papi, Agnieszka Dobrowolska, Iwona Krela-Kaźmierczak

**Affiliations:** ^1^Department of Gastroenterology, Dietetics and Internal Medicine, Poznan University of Medical Sciences, Poznan, Poland; ^2^Doctoral School, Poznan University of Medical Sciences, Poznan, Poland; ^3^Inflammatory Bowel Disease Unit, Department of Gastroenterology, S. Filippo Neri Hospital, Rome, Italy

**Keywords:** Mediterranean diet, inflammatory bowel diseases, gut microbiota, Crohn's disease, ulcerative colitis

## Abstract

Inflammatory bowel diseases (IBD) are chronic, progressive and relapsing inflammatory disorders of unknown etiology that may cause disability over time. Data from epidemiologic studies indicate that diet may play a role in the risk of developing and the course of IBD. It is known that the group of beneficial bacteria was reduced in the IBD and that the Mediterranean diet (MD)—which is defined as eating habits characterized by high consumption of plant foods, mainly cereals, vegetables, fruit as well as olive oil, and small portions of dairy products, sweets, sugar and meat products—affects gut microbiota, enriching beneficial bacteria, which support gut barrier function and reduce inflammation. Although several studies support different favorable effects of MD on IBD, adherence to MD by IBD patients is generally low, including patients from the Mediterranean Basin. Patients avoid many products which are elements of MD because there cause gastrointestinal symptoms. Patients should be encouraged to have a healthy and well-balanced diet according to individual tolerance of products. A good option seems to be good modified MD, changing hard-to-digest products to easy digest.

## Graphical Abstract

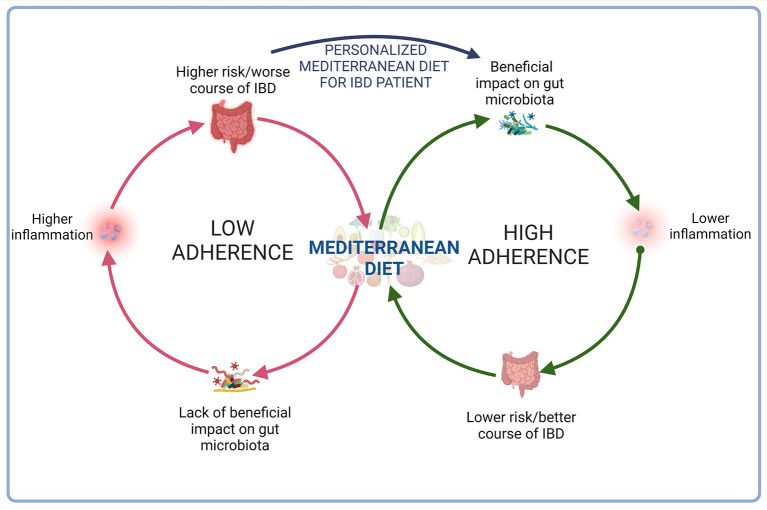

## 1. Introduction

The number of patients with inflammatory bowel diseases (IBD), including Crohn's disease (CD) and ulcerative colitis (UC), is rising globally (1). Many medications may modulate the course of the disease (2); however, looking for no pharmacological therapy for IBD seems to be a worth emphasizing element in improving patients' quality of life (QoL). One of these factors is diet. The study showed that the Western diet disturb gut microbiota and contains pro-inflammatory potential food. The opposite of the Western diet is the Mediterranean diet (MD), which basis are plant food, such us fruits, legumes, vegetables and whole grain food, as well as fish (2).

In this paper, we analyse the possible role of MD on IBD courses, offering a critical and comprehensive review of available evidences.

### 1.1. Inflammatory bowel diseases

IBD are disorders with chronic-relapsing course that may lead to disability. The etiology of IBD remains unknown: genetic and environmental factors, gut microbiota and immunological unbalance play a complex role in the pathogenesis (3–6).

IBD affect people in every age- from early childhood to late adulthood- even if, more than 80% of new cases are diagnosed in the second or third decade of life.

Nowadays an increasing prevalence of IBD is observed (7): in 1990, there were 3.7 million of people suffering from IBD around the world, and in 2017 they raised to 6.6 million (1). At a regional level, the highest age-standardized prevalence rate of IBD was observed in North America (422.0 cases per 100,000 population), while the lowest age-standardized prevalence rate was observed in the Caribbean (6.7 cases per 100,000 population). The incidence and prevalence of CD and UC appear to be lower in Asia and the Middle East; however, the frequency of IBD in some countries of Africa, Asia and South America, which were industrialized in last years, has also been rising (8). Furthermore, it seems to be a north-to-south gradient: higher incidence rates of CD and UC is observed in northern locations compared with southern latitudes (9). In fact, Jewish population presents higher frequency of IBD when compare with non-Jewish (10, 11), and the incidence is lower in Hispanic and Black populations than in White populations (10, 11). These, differences of geographical and ethnic are related to environmental and lifestyle factors as well as genetic factors. As an example, in a large Danish population-based study, the risk of IBD was lower in first-generation immigrants compared with individuals from Denmark (incidence rate ratio [IRR] 0.76, 95% CI 0.74–0.79) and the risk of IBD, however, was not significantly different for second-generation immigrants (11).

The most common symptoms of IBD patients, affecting QoL are abnormal bowel functions, rectal bleeding and abnominal pain.

About 50% of IBD patients have mild course of disease with low frequency of relapses and complications; on the other hand, the remaining patients may present a severe course with frequent relapses, need of hospitalisations, and evolutive behavior toward intestinal complications and need to surgical intervention (12, 13).

In UC, inflammation occurs in mucosa and submucosa of rectum and colon. The inflammation extends proximally and continuous. Proctitis (lesions are present only in the rectum) affects 30–35% of patients, left-sided colitis (lesions are present also in the left colon) affects 30–45% of patients and about 20–25% of patients suffer from extensive (also the right colon is involved by lesions). During disease course, the frequency of pancolitis may raise and affect ~50% of patients after 20 years of disease. During the course of the disease, in some patients, UC may be complicated by toxic megacolon, massive hemorrhage, colon perforation, colon-rectal cancer (12, 13).

In CD, inflammation is transmural and may affect various gastrointestinal tract sites. Ileocolonic disease is present in about 40% of CD patients at diagnosis, and both isolated ileal disease and isolate colonic disease in 30% for each localization. Upper gastrointestinal and perianal affected among 5–15% and 20–30% of patients, respectively (12, 13). Localization of disease is relatively constant, but the clinical manifestation is dynamic with many changes over time (14, 15). During follow-up, at least one-third of CD patients develop complications: intestinal strictures, internal or perianal fistulas or abscesses and are then classified as structuring or penetrating disease (15), and about ½ of CD patients have an aggressive disease course with more frequently exacerbation, hospitalizations and complications, which may lead often to surgery interventions (16).

The Montreal classification is commonly used to evaluate disease subtypes and their prognosis and to guide the challenge of choosing the most appropriate therapies for each disease subtype (17).

The goals of current treatment options (antibiotics, steroids, immunosuppressive drugs, biological therapies, small molecules) are to induce symptomatic remission, maintain steroid-free remission, enhance the QoL, prevent/treat complications of the disease avoiding short and long-term toxicity of therapy. Therapy should modify the course of the disease and prevent the disabling condition and irreversible tissue damage. Furthermore, therapy should be tailored according to patients' risk of developing disabling disease (18).

### 1.2. Mediterranean diet

Ancel Benjamin Keys introduced the term “Mediterranean diet” (MD) for the first time in the 1960s. His milestone study showed that the populations living along the Mediterranean Basin had a lower mortality rate and incidence of cardiovascular disease and cancer than other populations. MD thus define eating habits characterized by high consumption of cereals, vegetables, fruit, olive oil, and small portions of dairy products, pastry, sugar and meat products at the same time. Additionally, the intake of fish and wine is moderate. Although the Pyramid of Mediterranean lifestyle did not define the type of wine—white, red or rose—it is mainly red wine, containing more resveratrol than white wine (19, 20).

The pyramid of the Mediterranean diet was presented in 2011 by Mediterranean Diet Foundation. The components and portions of this diet are presented in Table 1 (21).

**Table 1:** Components of the Mediterranean lifestyle.

**Products**	**Serving in:**		
	**Every main meal**	**Every day**	**Every week**
Fruits	1–2		
Vegetables	≥2		
Oliva oil	1–2		
Bread/Pasta/Rice/ couscous/other cereals (preferably whole grain)	1–2		
Olives/nuts/seeds		1–2	
Dairy products (preferably low fat)		2	
White meat			2
Fish/seafood			≥2
Potatoes			≤ 3
Eggs			2–4
Legumes			≥2
Red meat			<2
Processed meat			≤ 1
Sweets			≤ 3

Additional elements of pyramids:

Herbs/spices/garlic/onions (less added salt) should be used daily.

The base of the pyramid is created by:

Regular physical activity.

Adequate reset.

Conviviality.

Choosing of biodiversity, seasonality, traditional, local and eco-friendly products.

Some diseases may be related to chronic inflammation and gut microbiota imbalance. According to meta-analysis, MD may reduce inflammation and positively affects gut microbiota (22). Additionally, systematic review reported that adherence of MD reduce biomarkers of inflammation and oxidative stress (23). Koloverou et al. (24) also reported that MD decreased systematic inflammation and increased total antioxidant capacity. Higher MD score was associated with decreased inflammatory markers—lower NF-κB; additionally, the adiponectin level was higher (25). Additionally, Mitjavila et al. (26) reported that MD protects lipids and DNA from oxidative damages in patients with the metabolic syndrome. Moreover, MD supplemented extra virgin olive oil and nuts affect serum nitric oxide (NO) concentration, endothelin-1 and endothelin-1 receptors, which influence on blood pressure and endothelial function (27). Mediterranean diet is especially important in cardiovascular disease prevention; however, many studies present a beneficial effect on other human body systems. According to a meta-analysis, Mediterranean diet adherence improved survival among people with cardiovascular disease (28). Moreover, MD reduce the risk of cardiovascular disease in patients with non-alcoholic fatty liver disease, metabolic syndrome and atrial fibrillation by lowering oxidative stress, improving antioxidant status, and decreasing insulin resistance (29). Patients with metabolic syndrome and higher adherence to MD presented lower alteration of anthropometric parameters and better oxidative and inflammatory status (30). However, MD does not decrease the risk of all-cause mortality among patients with a history of heart failure (31). It is vital to notice that also, interventions for MD improve endothelial function, which might prevent the atherosclerotic process (32). Urpi-Sarda et al. (33) reported that MD has an anti-inflammatory effect on the cardiovascular system. Moreover, MD reduces the risk of metabolic syndrome as well as components of this (waist circumference, high-density lipoprotein cholesterol level, triglycerides level, MD may also reduce the risk of cardiovascular disease, stroke, breast cancer (34) and lower systolic and diastolic blood pressure (35). Pintó et al. (36) reported that MD supplemented with extra virgin olive oil decrease risk of hepatic steatosis among elderly patients with high cardiovascular risk. Additionally, MD may decrease the risk of chronic kidney disease (37). The study showed that MD might also influence cognitive health. According to a meta-analysis, high adherence to the Mediterranean diet reduces the global cognitive decline risk in older adults without dementia (38). Moreover, Hill et al. (39) reported that MD has a small but significant effect on biomarkers of Alzheimer's disease. According to a systematic review, MD protects from Alzheimer's and Parkinson's disease (40). Based on the above evidence, MD is a dietary pattern that plays an important role in preventing many diseases, including inflammatory diseases and causes of mortality.

## 2. Mediterranean lifestyle and IBD

Data from epidemiologic studies suggest, also that dietary factors may play a role in the risk of developing IBD:

- Increased consumption of total fat, animal fat, and polyunsaturated fatty acids has been correlated with an increased incidence of IBD (41, 42);
- A high consumption of dietary fibers, particularly from fruit and cruciferous vegetables, has been linked with a decreased risk of CD (43);
- A high intake of omega-3 fatty acids as well as high intake of omega-6 fatty acids has been linked with a higher risk of developing CD (41);
- Intake of vitamin D is negatively associated with the risk of CD (whereas deficiency of vitamin D is common among IBD patients) (44–46).

Food antigens are thought to cause an immune system response leading to the development of intestinal inflammation; however, we do not know a specific pathogenic antigens, which are responsible for that (42). It is vital to notice that, the possible role of some dietary components, therapeutic enteral nutrition appears to be effective in CD, especially in children (47). In addition, smoking habits and some drugs (antibiotic, nonsteroidal anti-inflammatory, oral contraceptives, and hormone replacement) may increase the risk of developing IBD, but the magnitude of the risk appears small (5).

It is known that the group of beneficial bacteria was reduced in the IBD and that MD affects gut microbiota, enriching beneficial bacteria, which support gut barrier function and reduce inflammation (48). MD stimulates *Bifidobacteria, Lactobacilli, Eubacteria, Bacteroides*, and *Prevotella* (49). Among healthy adults, subjects with high MD adherence presented lower *Escherichia coli* counts and higher bifidobacteria/*E. coli* ratio (50). The data suggest, MD affect gut microbiota positively, decreasing risk of IBD (51). Mediterranean-like dietary pattern reduces intestinal inflammation among healthy first-degree relatives of Crohn's disease patients (52) and higher adherence to MD is associated with a lower risk of later-onset Crohn's disease (53). However, the study showed that only 1/4 of males and 1/5 of females with CD met the criteria of fruit consumption in the Mediterranean diet. Additionally, 21% of men and 32% of women consumed fish and/or shellfish three or more times per week. According to MD, over 70% of patients limited butter, beverages with added sugar, and red and processed meat (54). Although several studies support different favorable effects of MD on IBD (Table 2), adherence of MD by IBD patients is generally low, including patients from the Mediterranean Basin (Table 3).

**Table 2:** Impact of the Mediterranean diet on course of inflammatory bowel disease.

**Population**	**Outcome of the study and parameters used to measure the endpoint**	**Description of study**	**Conclusion**	**References**
100 children with mild to moderate IBD activity (age: 12–18 years)	Clinical scores (PCDAI and PUCAI) Serum CRP level, Fecal calprotectin, Serum TNF-level, Serum IL17 level, Serum IL10 level, Serum IL12 level, Serum IL23 level	Prospective, randomized study Children were divided into two groups: 1- Received MD diet 2- Received usual diet	Clinical scores and inflammatory markers among children and adolescents with IBD (active: mild-moderate) may be improved by MD PCDAI (*p* = 0.02) PUCAI (*p* = 0.04) Serum CRP level (*p* = 0.01) Fecal calprotectin (*p* = 0.03) Serum TNF- level (*p* = 0.04) Serum IL17 level (*p* = 0.02) Serum IL10 level (*p* = 0.1) Serum IL12 level (*p* = 0.02) Serum IL23 level (*p* = 0.03)	(77)
142 IBD patients in Italy	Liver steatosis	Prospective, interventional study	Disease activity and inflammatory markers level were improved	(70)
	Serum CRP level	CD and UC patients followed MD for 6 months	Reduced number of patients with active disease (*p* = 0.004 for UC patients and *p* = 0.011 for CD)	
	Fecal Calprotectin			
	CDAI			
	Partial Mayo score			
125 IBD children (aged: 5–17 years old) and 125 healthy children	Fecal Calprotectin	Cross sectional study	MD seems to correlate with reduced intestinal inflammation - significant differences in fecal calprotectin level (*p* = 0.027)	(78)
	Serum CRP level,	Diet was assessed based on a 3-day 24-recall and the Mediterranean Diet Quality Index for Children and Adolescents		
	PUCAI/PCDAI			
153 patients with ulcerative colitis after pouch surgery	Pouchitis disease activity index	Prospective observational study	Adherence to MD was associated with reduced fecal calprotectin level (OR = 0.74 [0.56–0.99])	(71)
	Serum CRP level	Adherence of MD was calculated based on the Mediterranean diet score		
	Fecal calprotectin			
86 outpatients with CD	Inflammatory bowel	Retrospective study	Adherence to MD was correlated with a higher quality of life (*p* = 0.00)	(72)
	Disease questionnaire	Adherence of MD was assessed using Mediterranean Diet Score		

PCDAI, pediatric Crohn's disease activity index; PUCAI, pediatric ulcerative colitis activity index; CRP, C-reactive protein; TNF, tumor necrosis factor; Il, interleukin; CDAI, crohn's disease activity index; MD, mediterranean diet; CD, crohn's disease; UC, ulcerative colitis; IBD, inflammatory bowel disease.

**Table 3:** Adherence to the Mediterranean diet by patients suffering from inflammatory bowel disease.

**Population**	**Outcome of the study and parameters used to measure the endpoint**	**Description of study**	**Conclusion**	**References**
100 outpatients of IBD in Australia	24-h diet recall and 17-point ready reckoner	Retrospective study	Low adherence of MD [MD adherence score was 5.1 ± 1.3 (max. 14 points)]	(79)
		Patients filled in questionnaires referring to diet		
80 IBD patients in Italy	Medi-Lite questionnaire	Retrospective study	MD adherence is dependent on disease activity in CD (*p* < 0.001)	(80)
	Short inflammatory bowel disease questionnaire	Patients filled in the Medi-Lite questionnaire and short inflammatory bowel disease questionnaire	Negative correlation between Medi-Lite and short inflammatory bowel disease questionnaire (*p* = 0.040)	
94 IBD patients in Croatia	Mediterranean diet service score (MDSS)	Cross-sectional study	Adherence to MD was very low [Average MDSS score was 6.0 (5.0–7.0); max. 24]	(81)
		Patients filled in Mediterranean Diet Service Score		

MD, mediterranean diet; IBD, inflammatory bowel disease.

MD also contains many bioactive compounds, such as polyphenols (55, 56), which present antioxidant activity (57). Moreover, consuming polyphenols improve epithelium cells' function and present an antioxidant effect (58). It is vital to notice that polyphenols modulate gut microbiota and promote the growth of lactobacilli and bifidobacterial. Gut microbiota changes may decrease gut inflammation (59). Sterniolo and Moreno reported that resveratrol, occurring mainly in red wine, may protect against colorectal cancer by inhibiting eicosanoids growth and Caco-2 growth (60).

Additionally, resveratrol metabolites may control apoptosis and cell cycle (61). *In vivo*, resveratrol also inhibits epithelial-mesenchymal transition (62). Another product rich in bioactive compounds is extra virgin olive oil—phenolic alcohols in them present chemoprotective activity. Additionally, oil polyphenols decrease inflammation and proliferation (63). Therefore, polyphenols may positively affect the course of IBD.

### 2.1. Mediterranean diet and course of IBD

Although a number of evidences, mainly coming from population-based observational studies, support a protective effect of MD against cardiovascular disease, stroke, metabolic disorders, several types of cancer, allergic diseases and Parkinson's and Alzheimer's disease (64–67), as state above, the impact of MD on IBD course remain unclear. Several experimental studies on epigenetic and transcriptomics analysis demonstrated that adherence to MD has a role in modulating the expression of inflammation-related genes and suggested a potential favorable anti-inflammatory effect (68, 69). More recently, the different studies from Italy, Greece and Israel, all of them conducted in the Mediterranean area, have linked the adherence to MD to an improvement of clinical and laboratory disease activity index in both CD and UC patients, suggesting a role of MD in reducing intestinal inflammation again (70–72).

However, to date, there is no evidence that MD, similarly to other alimentary regimens, can modify by itself disease course in IBD patients by reducing strong outcomes such as disease flares, hospitalisations, and need for additional treatments and surgery. Accordingly, ESPEN guidelines do not recommend to date any diet as a unique strategy for treating IBD patients (73).

## 3. Recommendations for the use of the Mediterranean diet in patients with IBD

The Mediterranean diet is high in vegetables, fruits, whole grains and nuts, and low in red and processed meat. However, data about the impact of MD on IBD are limited (74). However, MD is rich in fiber. Therefore, adherence to MD by IBD patients may be difficult, especially for patients with active disease. On the other hand, ESPEN does not recommend a specific diet as well as a high fiber diet during remission of IBD (75). Therefore, patients suffering from IBD can introduce MD in remission. However, they should observe gastrointestinal symptoms and eliminate products which worsen symptoms. Additionally, the Mediterranean diet meets the recommendation of the International Organization for the Study of Inflammatory Bowel Diseases (76). They recommend reducing red meat and myristic acid intake and increasing consumption of omega-3 fatty acids. According to these guidelines, CD patients should reduce saturated fatty acids consumption and increase their intake of fruits and vegetables. Finally, CD and UC patients should reduce the intake of emulsifiers, thickeners, maltodextrin, artificial sweeteners, and processed food containing titanium dioxide and sulphites. Certain food additives may increase intestinal permeability and higher inflammatory markers in gastrointestinal tissues (76) Avoiding trans-fatty acids and unpasteurised dairy products is recommended. Unpasteurised dairy products may cause infections, and trans-fatty acids increase lacking and inflammation (76). Patients should be encouraged to have a healthy and balanced diet according to individual tolerance of products. We want to propose some modifications to the Mediterranean diet for IBD patients:

1. IBD patients should choose easy-to-digest fruits and vegetables. They can peel them and cook lightly.
2. Patients must not choose whole grain bread, pasta, rice, couscous and other cereals. However, if they tolerated them well, choose it.
3. Nuts and seeds will be tolerated better after grinding.
4. Patients ought to avoid full fat as well as skimmed dairy products. Full fatty contains many saturated fatty acids, and skimmed are poor in fat-soluble vitamins and low-caloric. Semi-fatty will be the best option.
5. Hard-boiled, scrambled eggs or fried eggs may be hard to digest. Scrambled and fried eggs contain many fats, which make them harder to digest and cause long-time persist in stomach. Patients should choose soft-boiled eggs or poached eggs.
6. Patients should be careful during consuming legumes. Most of them are hard to digest, because are rich in fibers. The best option for IBD patients will be lentils, tempeh or tofu.

## 4. Summary

Mediterranean diet may be beneficial element, next to pharmacotherapy, influencing course of disease and lifestyle quality in IBD patient. In fact, MD reduces inflammation, and decreases also risk of other diseases, e.g., cardiovascular disease. Nevertheless, some products appearing in MD may be not tolerated by patients with gastrointestinal disorders, including inflammatory bowel diseases. For this reason, introducing MD to CD or UC patients, it should be paid attention on tolerance of this diet. MD should be personalized for each patients with the aim to reduce malaise and improving disease course.

## Author contributions

Conceptualization and supervision: IK-K. Writing—original draft preparation: AER, SF, AA, and CP. Writing—review and editing: AER, SF, AA, CP, AD, and IK-K. All authors have read and agreed to the published version of the manuscript.
